# Hippocampal expression of murine TNFα results in attenuation of amyloid deposition in vivo

**DOI:** 10.1186/1750-1326-6-16

**Published:** 2011-02-16

**Authors:** Paramita Chakrabarty, Amanda Herring, Carolina Ceballos-Diaz, Pritam Das, Todd E Golde

**Affiliations:** 1 Center for Translational Research in Neurodegenerative Disease, College of Medicine, University of Florida, 1275 Center Drive, Gainesville, PO Box #100159, FL-32610, USA; 2 Department of Neuroscience, Mayo Clinic College of Medicine, 4500 San Pablo Rd S, Jacksonville, FL-32224, USA

## Abstract

Fibrillar amyloid β (fAβ) peptide is the major component of Aβ plaques in the brains of Alzheimer's disease (AD) patients. Inflammatory mediators have previously been proposed to be drivers of Aβ pathology in AD patients by increasing amyloidogenic processing of APP and promoting Aβ accumulation, but recent data have shown that expression of various inflammatory cytokines attenuates Aβ pathology in mouse models. In an effort to further study the role of different inflammatory cytokines on Aβ pathology in vivo, we explored the effect of murine Tumor Necrosis Factor α (mTNFα) in regulating Aβ accumulation. Recombinant adeno-associated virus serotype 1 (AAV2/1) mediated expression of mTNFα in the hippocampus of 4 month old APP transgenic TgCRND8 mice resulted in significant reduction in hippocampal Aβ burden. No changes in APP levels or APP processing were observed in either mTNFα expressing APP transgenic mice or in non-transgenic littermates. Analysis of Aβ plaque burden in mTNFα expressing mice showed that even after substantial reduction compared to EGFP expressing age-matched controls, the Aβ plaque burden levels of the former do not decrease to the levels of 4 month old unmanipulated mice. Taken together, our data suggests that proinflammatory cytokine expression induced robust glial activation can attenuate plaque deposition. Whether such an enhanced microglial response actually clears preexisting deposits without causing bystander neurotoxicity remains an open question.

## Findings

Amyloid β (Aβ) plaques are a hallmark pathological feature of Alzheimer's disease (AD). Neuroinflammation, characterized by Aβ plaque associated reactive gliosis and increased levels of pro-inflammatory cytokines, has been hypothesized to lead to exacerbated AD pathology [1]. However, recent data has shown that activated glia [2-4] and myeloid lineage cells [5-8] ameliorate brain Aβ plaque load in Aβ precursor protein (APP) transgenic mouse models. We have previously demonstrated that increased expression of murine Interleukin-6 (IL-6) or murine Interferon-γ (IFNγ) results in attenuation of Aβ deposition in APP transgenic mice via synergistic activation of glia and innate immune system components [2,3]. In our effort to further analyze the role of different inflammatory cytokines on Aβ pathology, we investigated the effect of murine tumor necrosis factor α (mTNFα) expression in APP transgenic mice. TNFα is an inflammatory cytokine produced by macrophages, lymphoid cells, endothelial cells [9], neurons [10] and glia [11]. Various functional as well as genetic association studies have implicated increased levels of TNFα in exacerbating AD pathology [12]. Levels of TNFα and that of its receptors, TNF-RI and TNF-RII, are elevated in AD patients [13-15]. Though initial studies implicated three TNFα polymorphisms with increased AD [16], a subsequent study showed that these same polymorphisms delay the age of AD onset [17]. In preclinical mouse models, deletion of the TNF-RI gene or expression of the dominant negative truncated receptor reduces Aβ plaque formation in APP transgenic mice, suggesting a direct role in APP processing [18,19]. Studies in the 3 × Tg-AD mouse model expressing mutant APP, tau and presenilin showed that there was age-related increase in TNFα levels [20] and neuronal expression of TNFα exacerbated Aβ and tau pathology in these mice [21].

**Figure 1:**
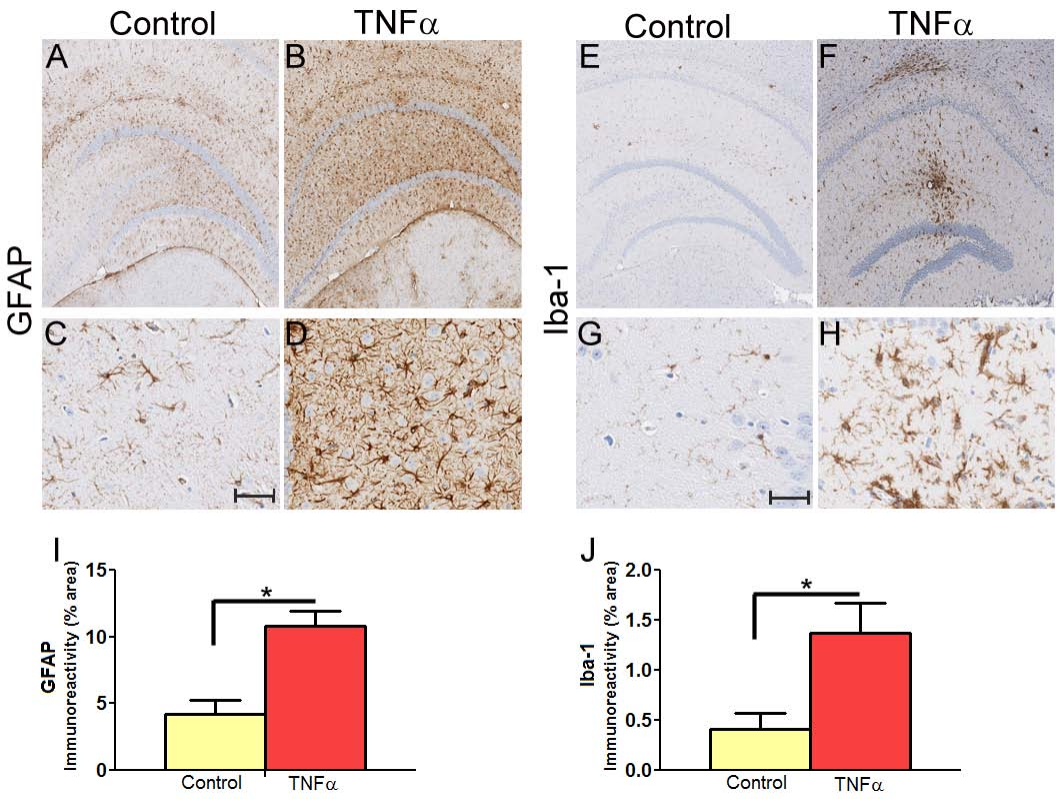
**AAV2/1 mediated expression of mTNFα in the hippocampus of TgCRND8 mice leads to robust neuroinflammation**. **A-D**. rAAV2/1-mTNFα or rAAV2/1-EGFP (Control) was injected into the hippocampus of 4 month old TgCRND8 mice and analyzed after 6 weeks. Representative images of GFAP immunoreactivity in paraffin embedded whole brain sections (A, B) and higher magnification of the hippocampus (C, D) of 5.5 month old TgCRND8 mice are shown. *Scale Bar*, 600 μm (A, B) and 25 μm (C, D). (n = 5/group). **E-H**. Representative images of Iba-1 immunoreactivity in paraffin embedded sections of 5.5 month old TgCRND8 mice injected with rAAV2/1 mTNFα or rAAV2/1-EGFP (Control). Whole brain sections (E, F) and the corresponding hippocampus (G, H) showing detailed morphology of the activated microglia is depicted. *Scale Bar*, 600 μm (E, F) and 25 μm (G, H). (n = 5/group). **I-J**. Quantitation of GFAP (I) and Iba-1(J) immunoreactivity burden (% area) in paraffin embedded sections of TgCRND8 mice expressing mTNFα or EGFP as control. The "positive pixel count" program available from Aperio was used for the analysis. (*p < 0.05; *t *test; n = 5/group).

To investigate the role of mTNFα in regulating Aβ accumulation in the CNS, we used recombinant adeno-associated virus (rAAV) to express mTNFα in the hippocampus of APP transgenic TgCRND8 mice [22]. Recombinant AAV2 plasmids containing mTNFα (Open Biosystems, clone 40126376) or enhanced green fluorescent protein (EGFP) under the control of the cytomegalovirus enhancer/chicken β actin promoter were packaged in AAV serotype 1 capsid (rAAV2/1) as described previously [2,23]. Adult 4 month old TgCRND8 mice were stereotaxically injected into the hippocampus (interaural coordinates, anteroposterior:-2.2, mediolateral:+/-1.6, dorsoventral: -1.2) with 2 μl of AAV2/1 constructs (1 × 10^13 ^particles/ml) and then sacrificed for analysis at 5.5 months (*n *= 5 for rAAV2/1-mTNFα; *n *= 6 for rAAV2/1-EGFP). The procedures were approved by the IACUC and performed as described previously [2]. In previous studies we have found that AAV2/1-EGFP expression has no effect on amyloid pathology or gliosis when compared to uninjected mice [2,23]; so, AAV2/1-EGFP injected mice were used as the control cohort. Following euthanasia, the mice brains were coronally dissected 1 mm anterior and 1 mm posterior to the point of injection and used for subsequent analysis. Anti-EGFP immunohistochemistry (Invitrogen, 1:1000) on paraffin embedded brain sections of AAV2/1-EGFP injected mice showed that the viral transgene is predominantly expressed in the hippocampal CA neurons, parts of the dentate gyrus, neuronal projections in the cortex and some overlying cortical neurons 6 week post-injection (Additional File 1, Fig S1, A-D). Reverse transcribed (Superscript III; Invitrogen) total RNA from mice brain was amplified using primer sequences from the Roche Universal Probe Library (Roche). Quantitative PCR analysis of transcripts showed that there were increased levels of mTNFα (Additional File 1, Fig S1, E) in the hippocampus of AAV2/1-mTNFα expressing mice compared to EGFP expressing controls. Immunohistochemical analysis performed on paraffin embedded mouse brain sections showed widespread GFAP positive astrocytes (anti-GFAP, Sigma, 1:1000; Figure 1A-D) as well as Iba-1 positive microglia (anti-Iba-1, Wako; 1:1000; Figure 1E-H) in the hippocampus of mTNFα expressing mice. Quantification of immunostaining using the "positive pixel count" program (Aperio, Vista, CA) showed significant increases (*p *= 0.05; *t *test) in both astrocytic staining (2.58 fold increase in GFAP burden; Figure 1I) and microglial staining (3.36 fold increase in Iba-1 burden; Figure 1J). Activated microglial immunoreactivity was especially robust along the projected path of stereotaxic injection (Figure 1F, H). Since TNFα has been shown to cause neuronal apoptosis [24], we performed hippocampal cell counts on paraffin embedded mouse brain sections using the "Nuclear Quantification" program (Aperio). Data from ten representative hippocampal CA areas (1000 μm × 60 μm) from each of three sections per sample (30 μm apart) were averaged for the final output (n = 4/group). No significant differences were found (*p *= 0.45, unpaired *t *test) in the number of hippocampal CA cells in the two mice groups (Additional File 2, Fig S2).

**Figure 2:**
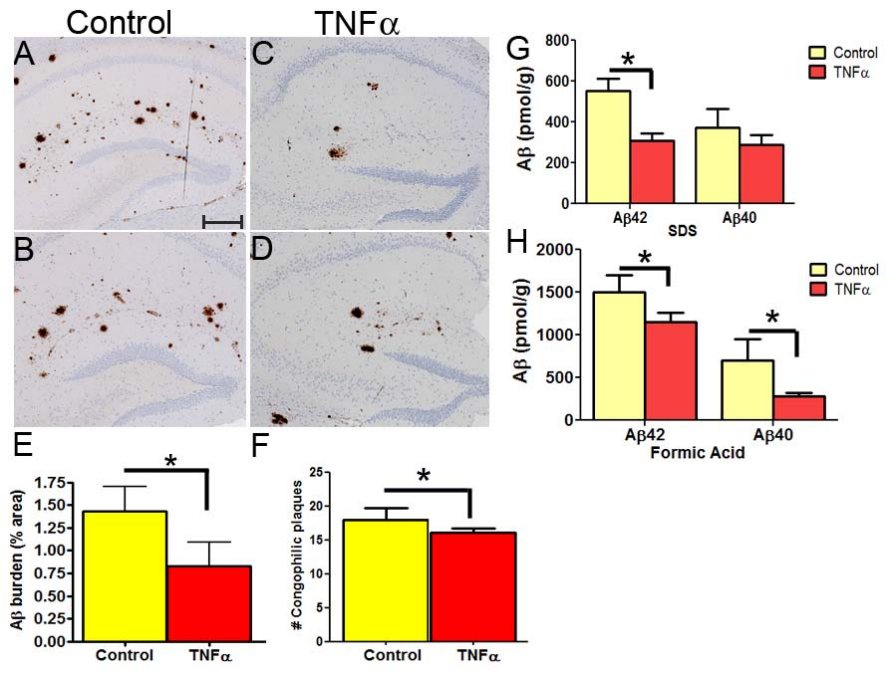
**Reduction of hippocampal Aβ in AAV2/1-mTNFα expressing TgCRND8 mice**. **A-D**. 4 month old TgCRND8 mice were stereotaxically injected in the hippocampus with either AAV2/1-mTNFα or AAV2/1-EGFP and sacrificed after 6 weeks (*n *= 5-6/group). Representative brain sections stained with 33.1.1 antibody (pan Aβ 1-16) depict attenuation of Aβ deposition in mTNFα expressing mice (C-D) compared to controls (A-B) in the immediate vicinity of the injection site. *Scale Bar*, 150 μm. **E**. Aβ plaque burden image analysis shows a significant decrease in amyloid deposition in mTNFα injected mice compared to control EGFP injected mice. At least three sections per sample, spaced 30 μm apart, were used for the analysis. (*p = 0.033; *t *test; n = 5/group). **F**. Quantitation of Congo Red stained compact Aβ plaques in mTNFα injected mice compared to control EGFP injected mice. Hippocampal compact plaques were counted from coronally dissected paraffin embedded representative brain sections of mTNFα and EGFP expressing mice. (*p = 0.05; *t *test; n = 5/group). **G-H**. Biochemical analyses of Aβ42 and Aβ40 levels by ELISA show significantly reduced SDS (G) and formic acid (H) extractable Aβ levels in mTNFα injected mice compared to controls (*p = 0.05; two way ANOVA; n = 4/group).

Aβ plaque burden in mice brain was quantified by immunohistochemistry with pan Aβ antibody 33.1.1 (1:1500, human Aβ1-16 specific), Aβ42 antibody 2.1.3 (1:1000; human Aβ42 specific) and Aβ40 antibody 13.1.1 (1:1000; human Aβ40 specific) [23]. Using the positive pixel count program (Aperio), the plaque burden (% area) was calculated by averaging the data obtained from three sections, 30 μm apart, per sample (n = 5-6/group). We observed a 36% (*p *= 0.033; *t *test) decrease in total Aβ plaque burden within the dissected coronal section in the mTNFα expressing mice compared to EGFP injected mice (Figure 2A-E). A small (10%; *p *= 0.05; *t *test) but statistically significant decrease in compact congophilic plaques was seen in the hippocampus of the mTNFα expressing mice compared to controls (Figure 2F). Biochemical levels of Aβ was measured following serial extraction of mouse hippocampus with RIPA buffer (Boston Biological), 2% SDS and 70% formic acid (FA) (n = 5-6/group). A significant reduction in SDS extractable Aβ42 (59%, p < 0.05, two way ANOVA) and a non-significant 23% reduction in Aβ40 levels in the mTNFα expressing mice compared with age-matched EGFP-expressing controls was noted (Figure 2G). Similarly, the FA fractions showed a 23% reduction in Aβ42 (*p *< 0.05; two way ANOVA) and a 43% reduction in Aβ40 levels (*p <*0.05; two way ANOVA) in rAAV2/1-mTNFα expressing mice compared to controls (Figure 2H). Janelsins et al [21] have reported increased intracellular Aβ following hippocampal expression of AAV-human TNFα in 3 × Tg-AD mice; although following long-term expression, there was decreased extracellular Aβ plaque deposition, which the authors attributed to neuronal loss. Though we have noticed sparse punctuate intracellular Aβ immunorectivity in CRND8 mice, primarily in a subset of neurons within the subiculum, such intracellular Aβ immunoreactivity was not notably altered by mTNFα (data not shown). The differences in our current observations may be due to the differences in transgenic mouse models and timing of experimental endpoints.

**Figure 3:**
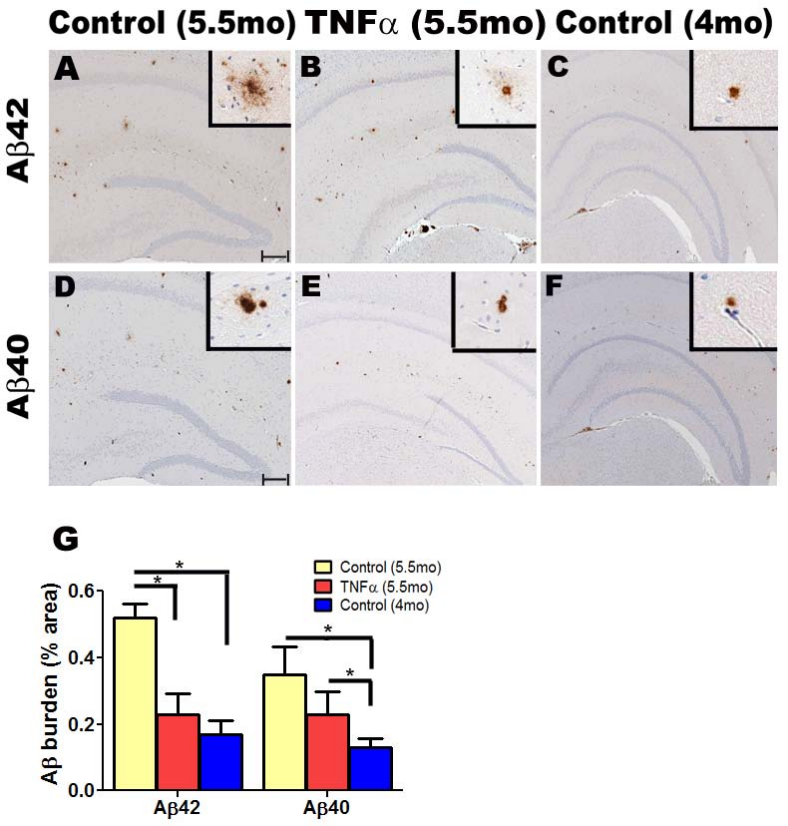
**Analysis of Aβ42 and Aβ40 plaque burden reveals that acute focal expression of mTNFα in the hippocampus of TgCRND8 mice results in attenuation of Aβ deposition**. **A-F**. 4 month old TgCRND8 mice were stereotaxically injected in the hippocampus with either rAAV2/1-mTNFα or rAAV2/1-EGFP and sacrificed after 6 weeks. Representative brain sections from EGFP and mTNFα expressing 5.5 month old mice as well as unmanipulated 4 month old mice were stained with anti Aβ42 antibody (A-C) and anti Aβ40 antibody (D-F). Insets depict representative magnified field of views from corresponding low power panel. *Scale Bar*, 600 μm (A-F); inset, 25 μm. (n = 5/group). **G**. Hippocampal Aβ42 and Aβ40 plaque burden analysis was performed in paraffin embedded brain sections of 5.5 month old mTNFα and EGFP expressing mice as well as unmanipulated 4 month old TgCRND8 mice. At least three sections per sample, spaced 30 μm apart, were used for the analysis. The "Positive Pixel Count" program available from Aperio was used for the analysis. (*p < 0.05; two way ANOVA; n = 5/group).

To probe whether the decrease in Aβ is the result of an active clearance process or a result of inhibition of deposition, we compared the extent of reduction of Aβ levels in mTNFα expressing mice to the Aβ levels in the original starting point of the study by analyzing the plaque burdens of 5.5 month old mTNFα expressing mice, 5.5 month old EGFP expressing mice (age-matched control) and 4 month old unmanipulated mice. We quantified both the "cored" Aβ plaque burden (indicated by Aβ40 specific 13.1.1 immunostaining; Figure 3D-F) and "total" Aβ plaque burden (indicated by Aβ42 specific 2.1.3 immunostaining; Figure 3A-C) in the hippocampus of 5.5 month old TgCRND8 mice and compared them to unmanipulated 4 month old TgCRND8 mice (n = 5/group). There were reductions in Aβ42 and Aβ40 plaque burden in the mTNFα expressing mice compared with age-matched EGFP-expressing controls (56%, *p *< 0.05, two way ANOVA, and 35%, p > 0.1; two way ANOVA, decrease respectively) (Figure 3G). However, both the Aβ42 and the Aβ40 plaque burdens of the unmanipulated 4 month old group was lower than the mTNFα-expressing mice, by 25% (p > 0.1; two way ANOVA) and 42% (*p *< 0.05; two way ANOVA) respectively (Figure 3G). The larger decrease in Aβ42 plaque burden, which measures both diffuse and cored plaque with a relatively smaller decrease in the Aβ40 "cored" plaque burden, suggests that mTNFα has preferential effects on more diffuse plaques.

**Figure 4:**
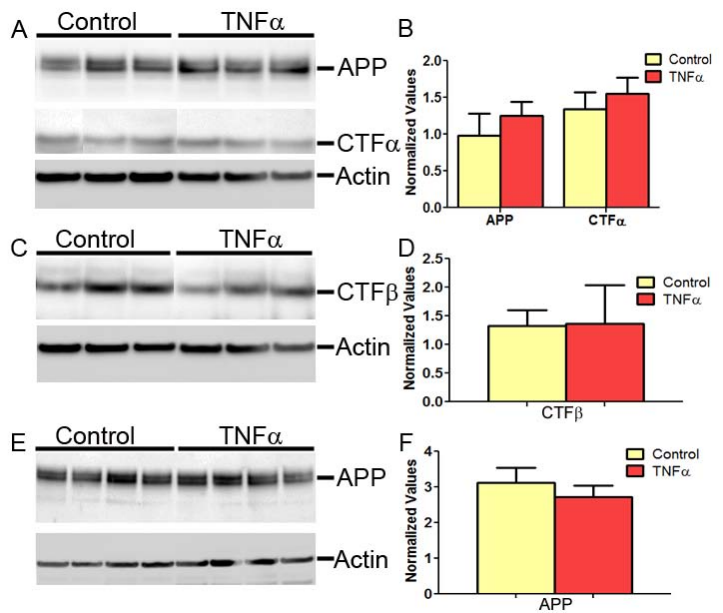
**APP or APP CTF levels were not significantly altered in mTNFα expressing mice**. **A-B**. Representative anti CT20 immunoblot showing no significant changes in APP or CTFα levels in AAV2/1-mTNFα expressing TgCRND8 compared to age-matched transgenic mice expressing EGFP (control cohort) (A). Intensity analysis of anti CT20 immunoreactive APP and CTFα levels was normalized to β-actin in TgCRND8 mice cohort (B). (n = 3/group). **C-D**. Representative anti 4G8 immunoblot showing no significant changes in CTFβ levels in AAV2/1-mTNFα expressing TgCRND8 compared to age-matched transgenic mice expressing EGFP (control cohort) (C). Intensity analysis of anti 4G8 immunoreactive CTFβ levels was normalized to β-actin in TgCRND8 mice cohort (D). (n = 3/group). **E-F**. Representative anti CT20 immunoblot showing no significant changes in APP levels in AAV2/1-mTNFα expressing non transgenic B6C3 mice compared to age-matched non transgenic B6C3 expressing EGFP (E). Intensity analysis of anti CT20 immunoreactive APP levels was normalized to β-actin in the wild type mice cohort (F). (n = 4/group).

To determine whether mTNFα alters APP levels or APP processing, we performed immunoblotting using anti APP CT20 antibody (1:1000) [2] and 4G8 antibody (Chemicon; 1:1000) on RIPA extracted brain lysates (n = 3/group). No significant changes in APP levels (Figure 4A-B) or APP C terminal fragments (CTFα and CTFβ) between mTNFα and EGFP expressing TgCRND8 mice were detected (Figure 4A-D). Additionally, no significant changes were detected in endogenous APP protein levels (n = 4/group) in mTNFα injected wild type B6C3 mice (non-transgenic littermates of TgCRND8 mice) compared to control age-matched EGFP expressing B6C3 mice (Figure 4E-F), signifying that mTNFα does not alter endogenous mouse APP expression through interaction with cellular transcriptional or post-transcriptional mechanisms. Since other pro-inflammatory cytokines, namely, Interleukin-6 [3] and Interferon γ [2] also fail to cause upregulation in APP levels in vivo, the possible involvement of a pro-inflammatory cytokine induced amyloidogenic "cytokine cycle" [25] in AD pathology is debatable.

**Figure 5:**
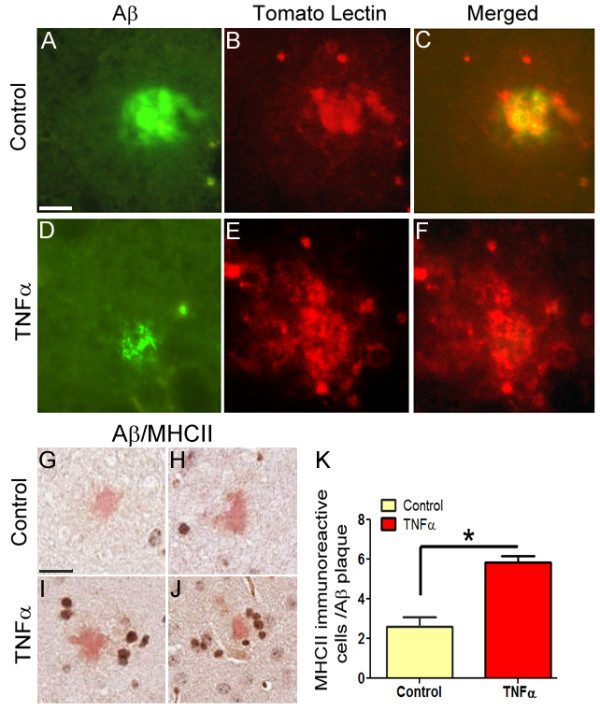
**Increased levels of immune activation in mTNFα expressing TgCRND8 mice**. **A-F**. Representative sections from the hippocampus of EGFP (A-C) or mTNFα expressing mice (D-F) from 5.5 month old TgCRND8 mice depicting increased clustering of tomato lectin binding activated glia (depicted in red) in close contact with Aβ plaques (depicted in green). Column marked "merged" depicts a merged dual color depiction of the corresponding tomato lectin and Aβ immunostaining. Scale Bar, 25 μm. (n = 3/group). **G-J**. Two representative sections from the hippocampus of EGFP (G-H) or mTNFα expressing mice (I-J) from 5.5 month old TgCRND8 mice depicting increased MHCII stained cells with the morphology of peripheral monocytes around cored plaques in mTNFα expressing animals. The sections have been counterstained with hematoxylin and Congo Red. Scale Bar, 25 μm. (n = 5/group). **K**. The number of MHCII immunopositive cells in the immediate proximity of Congophilic Aβ cored plaques were quantified by averaging from at least ten individual plaques from each sample. (*p < 0.05; *t *test; n = 5/group).

In the absence of changes in steady state APP levels, we investigated whether mTNFα-induced Aβ reduction results from enhanced activation of the innate immune system. To test for the presence of activated glia, we performed double-labeling immune-fluorescent staining using Texas Red conjugated Tomato Lectin (Vector Labs, 1:500) and 4G8 anti Aβ antibody (1:1000; Chemicon). The intensity and clustering of tomato lectin staining in close apposition to Aβ plaques was found to be increased in the hippocampus of mTNFα expressing mice (Figure 5D-F) compared to age-matched EGFP expressing controls (Figure 5A-C). This is similar to our previous observations that enhanced glial association with plaques as well as phagocytic activity may contribute to reductions in Aβ loads in APP transgenic mice [2,4,8,26].

Previous studies have shown that TNFα expression in the brain results in T cells infiltrating the CNS [9] and that following Aβ immunization, T cells may play a role in Aβ removal [27]. However, no CD3 (Abcam; 1:100) immunopositive T cells were detected in close proximity to the Aβ plaques (Additional File 3, Fig S3, B), though the antibody recognized T cells in paraffin embedded tonsil (Additional File 3, Fig S3, A). To investigate the glial subpopulations that effectively restrict plaque deposition, we performed MHC II (Abcam; 1:150) immunostaining on paraffin embedded brain sections followed by Congo Red and hematoxylin counterstaining (n = 5/group). At least 10 Congophilic plaques from the hippocampus of each sample were averaged for the final analysis. MHC class II, a classic marker of activated scavenger cells [28] was previously shown to be highly expressed among glia directly in contact with amyloid plaques, suggestive of increased phagocytic removal of Aβ [4]. A 56% (*p *< 0.05, *t *test) increase in the number of MHCII reactive cells associated with Congophilic plaques was seen in the hippocampus of mTNFα expressing mice compared to EGFP expressing mice (Figure 5G-K). Collectively, our data demonstrates that acute expression of an inflammatory cytokine attenuates Aβ pathology. Given that the common feature between IL-6, IFN-γ and TNFα expression in the brains of APP mice is robust glial activation and absence of effects on APP processing, such data suggests that pro-inflammatory cytokine driven gliosis primarily prevents Aβ deposition and/or enhances Aβ aggregate removal.

**Figure 6:**
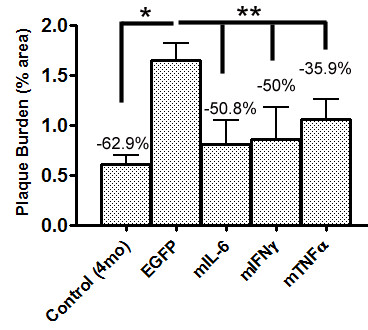
**Comparative analysis of Aβ plaque burden in TgCRND8 mice following acute hippocampal expression of pro-inflammatory cytokines**. 4 month old TgCRND8 mice were stereotaxically injected in the hippocampus with either rAAV2/1-IL-6, rAAV2/1-mIFNγ, rAAV2/1-mTNFα or rAAV2/1-EGFP and sacrificed after 6 weeks. Quantification of forebrain Aβ plaque burdens in 5.5 month old mice compared to unmanipulated 4 month old TgCRND8 mice is shown. (*p and **p = 0.0011; one way ANOVA; *n *= 5-6/group).

A contentious issue in this study and other similar studies [2] is whether pre-existing plaque pathology can be altered by modulating glial activity. This issue has important implications with respect to defining temporal windows for therapeutic intervention. Comparative analysis of Aβ plaque burdens from our previous studies [2,3] and the present study show that while there was a significant decrease in Aβ plaque burden in the cytokine expressing adult APP transgenic mice compared to control cohorts, the levels did not decrease beyond that of unmanipulated 4 month old mice (Figure 6; *p *= 0.0011, one way ANOVA). Thus, from our data, we can infer that following the expression of inflammatory cytokines in the brain and activation of innate immune system components, Aβ deposition is attenuated; however, whether active clearance may also be occurring concurrently is still debatable.

Using multiple paradigms, we and others [2-8] have demonstrated that proinflammatory cytokine mediated glial activation is associated with attenuation of CNS Aβ deposition in APP mouse models and in some cases possibly result in actual clearance of pre-existing plaques [29,30]. We find no evidence for altered levels or processing of the human APP transgene or endogenous mouse APP suggesting that enhanced glial activation attenuates Aβ accumulation most likely by altering Aβ clearance. Of course, the use of a human APP cDNA transgene driven by a heterologous promoter in many of these models imposes some limitations on the final interpretation of these studies, as it is possible that translational and transcriptional control of the APP gene and mRNA, respectively, is different between mice and humans. A next step could be to use the APP YAC transgenic mice [31], but even in this case the differences in the transcriptional and translational machineries of humans and mice may confound the outcome.

In AD patients and in AD mouse models there is an inevitable age-progressive accrual of Aβ aggregates despite widespread reactive gliosis. Thus, it appears that the normal reactive gliosis induced by Aβ accumulation is insufficient to overcome the inexorable accumulation of aggregated Aβ. Some have suggested that microglial aging may lead to inefficient or abortive Aβ phagocytosis [32,33], but such a hypothesis is difficult to reconcile with the observation that age of onset of Aβ plaque pathology in both humans and mouse models mice can occur very early in life. An intriguing possibility is that Aβ aggregates themselves impair glial function. Thus, as Aβ accumulates, glia-mediated clearance of Aβ aggregates decreases and such an effect could account for an apparent age-related effect in certain models. As shown here, in some circumstances it appears possible to modulate innate immune cell effector function in a manner that limits Aβ accumulation. Whether this modulation can be done safely remains an open question; balancing potential beneficial effects of innate immune cell activation in AD with potential neurotoxic consequences remains a formidable obstacle to development of therapies that involve manipulation of innate immunity.

## Abbreviations

(AAV): Adeno-associated virus; (AD): Alzheimer's disease; (Aβ): Amyloid β; (APP): Amyloid β precursor protein; (CTF): C-terminal fragment; (EGFP): Enhanced green fluorescent protein; (GFAP): Glial fibrillary acidic protein; (IACUC): Institutional animal care and use committee; (IL-1β):Interleukin-1β; (IL-6):Interleukin-6; 1 (Iba-1): Ionized calcium binding adaptor protein; (MHC II): Major histocompatibility complex II; (mTNFα): murine Tumor necrosis factor α; (3 × Tg-AD): triple transgenic mice.

## Competing interests

The authors declare that they have no competing interests.

## Authors' contributions

PC conducted the experiments and wrote the initial manuscript; AB performed amyloid burden analysis; CC-D prepared AAV2/1 viruses and quantitative PCR; PC, PD and TEG conceived the study and its design. PD and TG supervised the project and edited the manuscript preparation. All authors have read and approved the manuscript.

## Supplementary Material

Additional file 1 **Figure S1: AAV2/1 mediated expression of transgene in mice hippocampus**. **A-D**. AAV2/1-EGFP was stereotactically injected into the hippocampus of 4 month old TgCRND8 mice and analyzed after 6 weeks. Representative images of EGFP immunoreactivity on paraffin embedded whole brain sections (A, B) and the hippocampus (C, D) of EGFP injected or uninjected mice are shown. *Scale Bar*, 600 μm (A, B) and 25 μm (C, D). (n = 6/group). **E**. Expression of mTNFα was determined in 5.5 month old mTNFα expressing TgCRND8 mice compared to EGFP expressing age-matched transgenic controls using real time Q-PCR. Relative quantitation of mRNA transcript levels was performed using the comparative cycle threshold method. β-actin was used to normalize expression levels from the samples. Data, expressed as relative units of mRNA expression, represents averaged fold change values obtained from mTNFα expressing mice, relative to averaged values obtained from EGFP expressing mice. (n = 3/group, *p < 0.05). Click here for file

Additional file 2 **Figure S2. No significant changes in hippocampal CA neurons following acute hippocampal expression of rAAV2/1-mTNFα**. Quantification of cell count of the hippocampal pyramidal cells (CA1, CA2 and CA3) in TgCRND8 mice expressing mTNFα compared to controls is depicted. Data from three sections from each sample, spaced 30 μm apart, were averaged using the Aperio "nuclear quantification" program for the final output. (n = 4/group). Click here for file

Additional file 3 **Figure S3. No evidence of T cell accumulation around hippocampal Aβ plaques in rAAV2/1-mTNFα expressing animals**. Representative sections depicting CD3 immunostaining in tonsil (A) and 5.5 month old TgCRND8 mice injected with rAAV2/1-mTNFα in the hippocampus at 4 months of age (B). There is copious amounts of CD3 immunostained T cells in the tonsil (A and inset). Though we noticed some CD3 immunopositivity in the ventricles of 5.5 month old mTNFα expressing TgCRND8 mice (B and 2), we did not observe any T cell staining around Aβ plaques (asterisk "*" mark) in the hippocampus of these mice (B and 1). *Scale Bar*, 60 μm (A) and 25 μm (inset), 600 μm (B) and 25 μm (1, 2). (n = 4/group). Click here for file
