# Supplementary figures and images for: Hippocampal expression of murine TNFα results in attenuation of amyloid deposition in vivo
Source: Mol Neurodegener. 2011 Feb 16;6:16. doi: 10.1186/1750-1326-6-16 (PMC3050766; doi:10.1186/1750-1326-6-16)

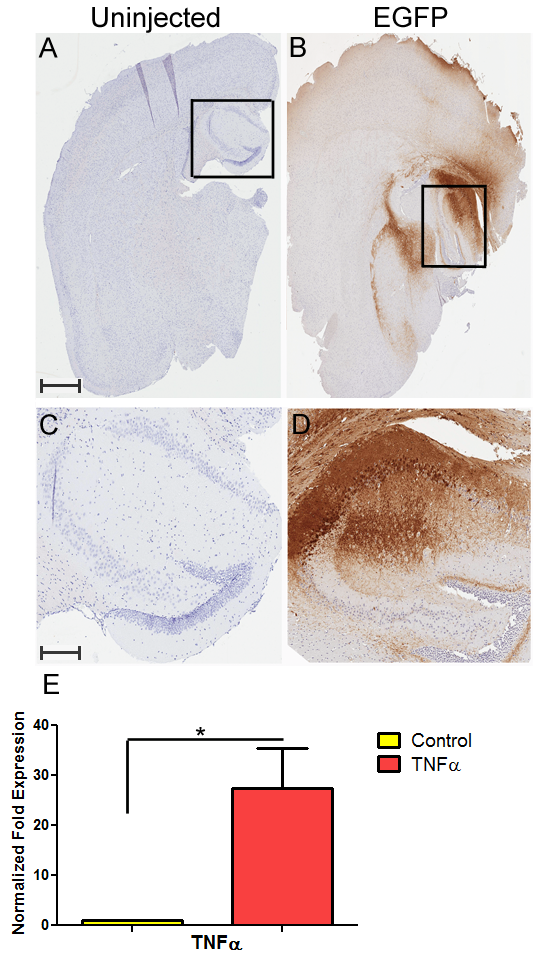

Supplement: Additional file 1 — Figure S1: AAV2/1 mediated expression of transgene in mice hippocampus. A-D. AAV2/1-EGFP was stereotactically injected into the hippocampus of 4 month old TgCRND8 mice and analyzed after 6 weeks. Representative images of EGFP immunoreactivity on paraffin embedded whole brain sections (A, B) and the hippocampus (C, D) of EGFP injected or uninjected mice are shown. Scale Bar, 600 μm (A, B) and 25 μm (C, D). (n = 6/group). E. Expression of mTNFα was determined in 5.5 month old mTNFα expressing TgCRND8 mice compared to EGFP expressing age-matched transgenic controls using real time Q-PCR. Relative quantitation of mRNA transcript levels was performed using the comparative cycle threshold method. β-actin was used to normalize expression levels from the samples. Data, expressed as relative units of mRNA expression, represents averaged fold change values obtained from mTNFα expressing mice, relative to averaged values obtained from EGFP expressing mice. (n = 3/group, *p < 0.05). [file 1750-1326-6-16-S1.TIFF]

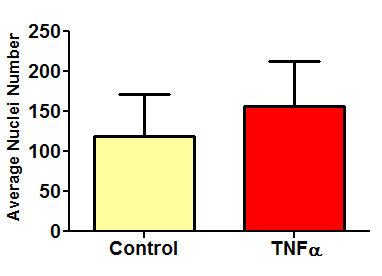

Supplement: Additional file 2 — Figure S2. No significant changes in hippocampal CA neurons following acute hippocampal expression of rAAV2/1-mTNFα. Quantification of cell count of the hippocampal pyramidal cells (CA1, CA2 and CA3) in TgCRND8 mice expressing mTNFα compared to controls is depicted. Data from three sections from each sample, spaced 30 μm apart, were averaged using the Aperio "nuclear quantification" program for the final output. (n = 4/group). [file 1750-1326-6-16-S2.TIFF]

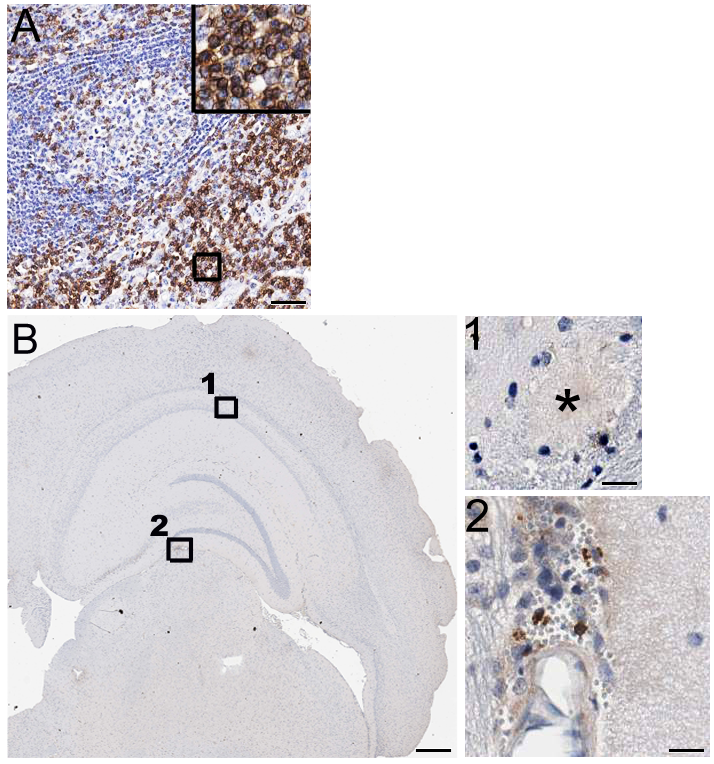

Supplement: Additional file 3 — Figure S3. No evidence of T cell accumulation around hippocampal Aβ plaques in rAAV2/1-mTNFα expressing animals. Representative sections depicting CD3 immunostaining in tonsil (A) and 5.5 month old TgCRND8 mice injected with rAAV2/1-mTNFα in the hippocampus at 4 months of age (B). There is copious amounts of CD3 immunostained T cells in the tonsil (A and inset). Though we noticed some CD3 immunopositivity in the ventricles of 5.5 month old mTNFα expressing TgCRND8 mice (B and 2), we did not observe any T cell staining around Aβ plaques (asterisk "*" mark) in the hippocampus of these mice (B and 1). Scale Bar, 60 μm (A) and 25 μm (inset), 600 μm (B) and 25 μm (1, 2). (n = 4/group). [file 1750-1326-6-16-S3.TIFF]
